# Successful treatment of refractory prurigo nodularis with abrocitinib

**DOI:** 10.1002/ccr3.8606

**Published:** 2024-03-07

**Authors:** Fang Sun, Zhenzhen Wu

**Affiliations:** ^1^ Derpartment of Dermatology Affiliated AoYang Hospital of Jiangsu University Zhangjiagang Jiangsu China

**Keywords:** abrocitinib, itching, JAK inhibitor, prurigo nodularis

## Abstract

Prurigo nodularis is frequently difficult to manage with conventional therapy. Given the pathogenesis and refractory nature, we demonstrate a case in which inhibition of JAK–STAT signaling may significantly improve prurigo nodularis. Based on the results, we would like to draw a conclusion that abrocitinib as an inhibitor of Jak is a promising choice for the treatment of prurigo nodularis.

Prurigo nodularis (PN) is a chronic skin disease that manifests with severe itchy, firm, and hyperkeratotic nodules distributed on the trunk and the extremities symmetrically. The treatment of prurigo nodularis (PN) has always been a great challenge for dermatologist. Here, we report a case of refractory PN successfully treated with selective Janus Kinase 1 (JAK1) inhibitor abrocitinib.

A 46‐year‐old man presented with visible dark brown nodules on the face, ears, hands, and back neck for 2 years (Figure 1). The patient complained of itchy skin all day especially at night that affects his night sleeping quality heavily. More important it occurs on visible sites such as the face and hands, which has profound psychosocial impacts on him. Laboratory evaluation of routine blood test, thyroid function, liver, and kidney function tests, HIV, syphilis test, and the serum total IgE level showed normal. The patient denied a personal history of allergic rhinitis, asthma, infantile eczema, and other systemic disease, and also denied a family history of allergies and photosensitivity.

**FIGURE 1:**
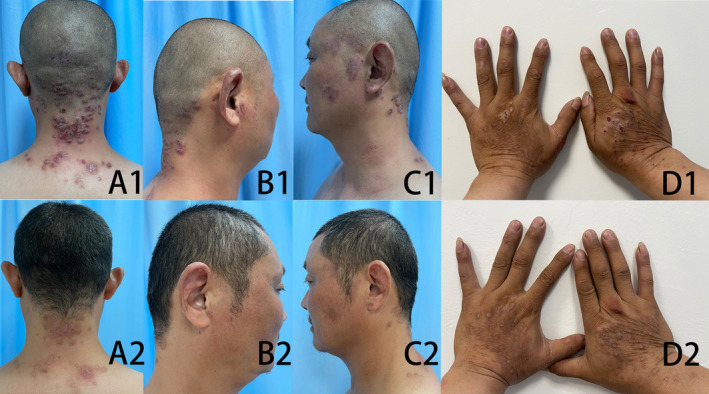
Clinical images of the back neck, ears, and hands at Week 0 (A1–D1) and after abrocitinib treatments at Week 8 (A2–D2).

Therefore, a diagnosis of non‐atopic PN was made. At first, we gave oral prednisolone 30 mg daily and compound clobetasol propionate ointment twice daily. After 1‐month treatment, Visual Analogue Scale (VAS) decreases form 9 to 3. The 2‐month prednisolone dose is given 20 mg daily. After 2 month above treatment, the itching was obviously relieved. But the patient gain 5Kg, especially the facial swelling is obvious. We have to stop the treatment. Therefore, thalidomide 50 mg twice daily was prescribed concomitantly with high potency topical corticosteroids. Unfortunately, the treatment was interrupted again due to dizziness. Because the patient is a driver, he cannot concentrating on driving. As a result, the lesions aggravated with even more severe pruritus and VAS rising to 10. The patient refused to use those medicines with strong side effects but could not bear expenses of dupilumab. Therefore, abrocitinib 100 mg daily was prescribed. The pruritus markedly improved in 3 days with peak VAS decreases from 10 to 3. Sustained effectiveness was achieved after 2 month treated with only a few nodules and mostly pigmentation on above area. Abrocitinib treatment was well tolerated.

Although the pathogenesis of PN is unknown, the sensation of the neural transmission of itch involves cutaneous nerve fibers in the skin to the dorsal‐root ganglion, spinal cord, and brain. Interleukin‐31 has been regarded as an itch cytokine; in multiple chronic pruritic skin diseases, it up‐regulated in the blood and can induce additional proinflammatory cytokines. Interleukin‐31 also induces phosphorylation of JAK1 and JAK2, which are known mediators of the itch signaling cascade.^2^

Abrocitinib is an oral small molecule inhibitor of Janus kinase 1(JAK1) for the treatment of moderate‐to‐severe atopic dermatitis(AD). The reported success cases of tofacitinib^1^ and baricitinib^3^ for PN both demonstrate the effectiveness of the Jak inhibitor for the treatment of PN. So we choose abrocitinib to treat this patient, it has also shown great efficacy.

Therefore, abrocitinib as an inhibitor of Jak is a promising choice for the treatment of PN, especially those patients who are resistant to conventional treatments or cannot afford dupilumab.

## AUTHOR CONTRIBUTIONS

**Fang Sun:** Conceptualization; data curation; visualization; writing – original draft. **Zhenzhen Wu:** Conceptualization; investigation; resources; writing – review and editing.

## CONFLICT OF INTEREST STATEMENT

The authors declared that they have no conflicts of interest to this work. We declare that we do not have any commercial or associative interest that represents a conflict of interest in connection with the work submitted.

## CONSENT

Written informed consent was obtained from the patient to published this report in accordance with the journal's patient consent policy.

## Data Availability

Data sharing is not applicable to this article as no new data were created or analyzed in this study.
